# GSK3β rephosphorylation rescues ALPL deficiency-induced impairment of odontoblastic differentiation of DPSCs

**DOI:** 10.1186/s13287-021-02235-7

**Published:** 2021-04-06

**Authors:** Liqiang Zhang, Jiangdong Zhao, Jiayi Dong, Yuting Liu, Kun Xuan, Wenjia Liu

**Affiliations:** 1 National & Local Joint Engineering Research Center of Biodiagnosis and Biotherapy, Precision Medicine Institute, The Second Affiliated Hospital of Xi’an Jiaotong University, No.157 Xiwu Road, Xi’an, 710004 China; 2 Xi’an Institute of Tissue Engineering and Regenerative Medicine, Xi’an, 710032 Shaanxi China; 3 The Key Laboratory of Aerospace Medicine, Ministry of Education, Air Force Medical University, Xi’an, 710032 Shaanxi China; 4 State Key Laboratory of Military Stomatology & National Clinical Research Center for Oral Diseases & Shaanxi International Joint Research Center for Oral Diseases, Center for Tissue Engineering, School of Stomatology, The Fourth Military Medical University, No. 145 West Changle Road, Xi’an, 710032 Shaanxi China; 5 Department of Pediatric Dentistry, School of Stomatology, Fourth Military Medical University, Xi’an, China

**Keywords:** Hypophosphatasia, Tooth defects, Odontoblastic differentiation, ALPL, DPSCs, GSK3β

## Abstract

**Background:**

Premature exfoliation of the deciduous teeth is a common manifestation in childhood patients with hypophosphatasia (HPP), which is an autosomal inherited disease caused by ALPL mutations. Dysplasia of the cementum, dentin, and alveolar bone has been proposed to be the main reasons for the exfoliation of teeth, while the extraordinarily complex intracellular mechanisms remain elusive. Dental pulp stem cells (DPSCs) have been demonstrated to successfully regenerate functional pulp-dentin-like tissue. Dental pulp cells derived from HPP patients impaired mineralization; however, insight into the deeper mechanism is still unclear.

**Methods:**

The effects of ALPL on odontoblastic differentiation of DPSCs from HPP patient were assessed by Alizarin Red staining, immunofluorescent staining, Western blot and RT-PCR, and micro-CT assays.

**Result:**

Here, we found DPSCs from HPP patient exhibited low ALP activity and impaired odontoblastic differentiation. Meanwhile, we found that loss of function of ALPL reduced phosphorylation of GSK3β in DPSCs. While GSK3β rephosphorylation improved odontoblastic differentiation of HPP DPSCs with LiCl treatment. Finally, we demonstrated systemic LiCl injection ameliorated tooth-associated defects in ALPL^+/−^ mice by enhanced phosphorylation of GSK3β in the teeth.

**Conclusions:**

Our study indicates that ALPL regulates odontoblastic differentiation of DPSCs and provides useful information for understanding how ALPL deficiency led to tooth dysplasia and, ultimately, may inform efforts at improvement tooth defects in HPP patients.

**Supplementary Information:**

The online version contains supplementary material available at 10.1186/s13287-021-02235-7.

## Background

Hypophosphatasia (HPP) is an inherited disease characterized with insufficient mineralization of the teeth and skeletal system caused by gene mutations of liver/bone/kidney-type alkaline phosphatase (ALPL), which encoded tissue-nonspecific alkaline phosphatase (TNAP) and leading to reduce activity of the enzyme [1–3]. The clinical manifestations of patients with HPP are a broad range from life-threatening in infantile or death in utero to mild forms in childhood or adult with only dental or skeletal symptoms. Accumulating clinical trials suggest that asfotase alfa (Strensiq™), a first-in-class drug was permitted to use for therapy for patients with HPP, and treatment can significantly improve the outcome for the life-threatened patients with HPP [4–6]. However, several problems remain to be worked out, despite asfotase alfa exhibited a significant improvement on bone mineralization. Hypersensitive and ectopic calcification in injection sites, pneumonia, and even severe chronic hepatitis or failed treatment have been reported [7–9], and the longer-time effects of asfotase alfa therapy in these HPP patients are still unknown.

Premature exfoliation of the deciduous teeth is the most common manifestation in childhood patients with HPP and severely affects the quality of life for the patients. Asfotase alfa on the improvement tooth defects in HPP patients was few reported. In addition, the expensive cost should also be considered, especially in economically under-developed areas. Thus, cheaper and more effective strategies need to be developed based on discovering molecular mechanism for treatment patients with HPP, especially in the mild forms of childhood- or odonto-HPP. Dysplasia or aplasia of the cementum, dentin, and alveolar bone was frequently observed in patients with HPP [10–12] and have been proposed to be the main reasons for the exfoliation of the teeth; however, the complex cellular mechanisms remain elusive.

Dental pulp stem cells (DPSCs) have been identified to be a population of capable of multilineage differentiation into a variety of cell types, including neurons, adipocytes, osteoblasts, and odontoblasts [13]. Strong evidences have demonstrated that DPSCs with tooth slice or root canal-scaffold could regenerate pulp-dentin like complex tissues when transplanted into subcutaneous of immunocompromised mice [14, 15]. Notably, our previous work showed that implanted autologous DPSCs aggregates into necrotic immature permanent incisors of pediatric patients successfully regenerated functional dental pulp with odontoblast layer [16]. These evidences suggest that DPSCs display excellent regenerative ability, which is important for tooth development and regeneration. Despite some studies have showed that dental pulp cells derived from HPP patients reduced ALP activity and actually impaired mineralization [17, 18], insight into the deeper mechanism remains unclear.

Canonical Wnt/β-catenin pathway is a key regulator for controlling mesenchymal stem cell (MSC) differentiation into osteoblasts/odontoblasts [19, 20]. Previous studies demonstrated that LiCl, an agonist of the canonical Wnt signaling by increased phosphorylation of GSK3β at Ser9 [20, 21], effectively stabilizes free cytosolic β-catenin leading to β-catenin translocation into nucleus. These reports showed that LiCl drives the transcription of target genes of canonical Wnt pathway, such as Runx2 and ALPL, and thereby enhanced MSC differentiation into osteoblasts [20, 21]. We have recently demonstrated that ALPL mutation impaired osteogenic differentiation of BMSCs due to impede activation of Wnt/ β-catenin pathway [22]. Summary, these studies largely implied that there is a feasible regulatory loop between ALPL and canonical Wnt pathways.

In this study, tooth defects were observed both in HPP patients and ALPL^+/−^ mice. In addition, DPSCs from HPP patient (HPP DPSCs) displayed low ALP activity and impaired lineage differentiation into odontoblasts. Subsequently, we found that loss function of ALPL inhibited activation of canonical Wnt pathway. While reactivating of canonical Wnt pathway promoted odontoblastic differentiation of HPP DPSCs with LiCl treatment. Finally, we demonstrated systemic LiCl injection ameliorated insufficient mineralization of tooth and mandible bone, and also improved exposure of collagen fiber network on the surface of dentinal tubules in ALPL^+/−^ mice. This work provides useful information for understanding tooth associated disorders caused by loss of function of ALPL and, ultimately, may inform efforts at improvement tooth defects in HPP patients.

## Methods

### Animals

Female ALPL^+/−^ mice (B6.129S7-Akp2tm1Sor/J, pure C57BL/6 J genetic background) were purchased from Jackson labs (Bar Harbor, USA) and maintained in specific pathogen-free conditions at 24 °C with a 12-h light/dark cycle. All of the procedures that involved animals were approved by the Animal Ethics Committee of the Xi’an Jiaotong University. Primers used for genotyping include: mutant-5′CCGTGCATCTGCCAGTTTGAGGGGA3′, wild type-5′CTGGCACAAAAGAGTTGGTAAGGCAG3′, common-5′GATCGGAACGTCAATTAACGTCAAT3′.

### Isolation and culture of DPSCs

Tooth samples were collected from healthy teeth extracted for orthodontic reasons and one patient with HPP (Dental Clinic of the Fourth Military Medical University, Xi’an, China). The clinical study was approved by the Ethics Committee of the Stomatology Hospital of the Fourth Military Medical University, and written informed consent was obtained from all participants prior to use tooth samples. To isolate dental pulp stem cells (DPSCs), dental pulp was enzymatically digested with type I collagenase (0.66 mg/ml; Sigma, USA) for 30 min, and single-cell deposits were suspended and cultured in а-MEM (Gibco, USA) supplemented with 10% FBS, 2 mM l-glutamine (Invitrogen, USA), 100 U/ml penicillin and 100 mg/ml streptomycin (Gibco) at 37 °C of 5% CO_2_.

### Alkaline phosphatase activity assay

To analyze alkaline phosphatase activity, the total proteins of DPSCs from normal and HPP patient were isolated and examined using an alkaline phosphatase assay kit from NJJCBIO company (NJJCBIO, A059-2-2, China) according to the manufacturer’s instructions. ALP activity was corrected with total proteins and ultimately was present as U/g.

### Colony-forming unit (CFU) assays

To assess the capacity and efficiency of cell self-renewal, 3 × 10^3^ DPSCs at passage 2 were seeded in a 5-cm plastic dish with 3 mL complete medium, and the medium was refreshed every 3 days for 2 weeks. On day 14, cells were washed with PBS, fixed by 4% paraformaldehyde, and stained with 0.5% toluidine blue for 30 min. Then, cells were washed with ddH_2_O until the dye stopped coming off. When a colony contains more than 50 cells were counted for one CFU, and the number of CFU in the dish was counted.

### Flow cytometry analysis

For identification of DPSCs phenotype, 3 × 10E5 DPSCs (passage 3) were incubated with PE- or FITC-conjugated monoclonal antibodies against human CD29, CD44, CD73, CD90, CD105, CD34, CD45 (eBioscience, CA), and Stro-1 (R&D Systems, MN) (sFig. 1), and flow cytometric analysis were performed using a Beckman CytoFLEX S flow cytometer (Beckman Coulter, USA).

### Cell proliferation and apoptosis assay

To analyze cell proliferation or cell apoptosis of DPSCs, cell cycle and apoptosis analysis were performed by flow cytometric analysis. For cell proliferation assays, DPSCs cultured in 25 cm^2^ culture flask with 0.1% a-MEM medium for 12 h and then replaced and cultured with 10% a-MEM medium for 24 h. Subsequently, the cells were digested with 0.25% trypsin and collected, fixed in precooling 70% ethanol overnight at 4 °C, and cell cycle tests were performed with a Cell Cycle and Apoptosis Analysis Kit (Yeasen, 40301ES60, China) according to the manufacture’s instruction. For cell apoptosis assays, the cells were digested with 0.25% trypsin and collected, and cell apoptosis tests were performed with a Annexin V-FITC/PI Apoptosis Detection Kit (Yeasen, 40302ES60, China) according to the manufacture’s instruction.

### DPSCs induced odontoblastic differentiation

DPSCs were incubated in odontoblastic medium (100 nM dexamethasone, 50 mg/ml ascorbic acid, and 5 mM β-glycerophosphate) for 3 weeks. To assess odontoblastic differentiation, the expression of DSPP and DMP1 were detected by real-time polymerase chain reaction (RT-PCR) and Western blotting after induced in odontoblastic medium 1 week. To analyze the mineralized nodes, the cells were stained with 1% Alizarin Red (Sigma, USA) after induced in odontoblastic medium 3 weeks. The mineralized nodules were dissolved with cetylpyridinium chloride monohydrate, and quantitative parameters were measured by spectrophotometer at 570 nm.

### Scan electron microscopy (SEM) assay

The human teeth, or molars from wild-type or ALPL^+/−^ mice, were fixed in 2.5% glutaraldehyde overnight at 4 °C, washed with 10% sodium hypochlorite in ultrasonic cleaning instrument for 30 min. Then, the samples were dried, coated with gold, and were imaged with a scanning electron microscope (SEM, S-3400 N) at a voltage of 5.0 kV. All measurements were made using the Cell Sense morphometric program (Olympus).

### Real-time PCR (RT-PCR) analysis

Total DPSC RNAs were isolated and extracted by RNAiso plus (TaKaRa, Japan) according to the manufacture’s instruction. Then, the mRNA was reversely transcribed into cDNA by Primescript™ RT master mix (TaKaRa, RR036A, Japan). Real-time PCR was performed with TB Green® Premix Ex Taq™ GC (TaKaRa, RR071A, Japan) and detected by CFX96 Trademark Real-time PCR detection system (Bio-rad, USA). Expression levels of DSPP, DMP1, and β-catenin were examined. All the RT-PCR data are presented as mean ± s.d. for triplicate samples from a representative experiment (*n* = 3). The primers used in real-time PCR were listed in the supplementary Table 1.

### Western blotting analysis

Total proteins from DPSCs were harvested with RIPA lysis buffer (Beyotime, China). The proteins were separated in sodium dodecyl sulfonate-polyacrylamide gels (SDS-PAGE), transferred to PVDF membranes (Millipore, USA), blocked in TBST containing 5% BSA, and then incubated in first antibodies with ALPL (Abcam, ab108337, 1:1000), DSPP (Santa Cruz Biotechnology, sc-73632, 1:500), DMP1 (Santa Cruz Biotechnology, sc-6551, 1:500), total GSK3β (Cell signaling technology, 9832, 1:1000), p-GSK3β (cell signaling technology, 9323, 1:1000), β-catenin (cell signaling technology, 8480s, 1:1000), active β-catenin (Millipore, 05-665, 1:1000), p-AMPK (cell signaling technology, 2535, 1:1000), p-ERK (cell signaling technology, 9101s, 1:1000), Gli1 (Novus Biological, NBP1-78259), TGFβ (Santa Cruz Biotechnology, sc-398, 1:500), and GAPDH (Cwbiotech, CW0100, 1:4000), respectively. Next day, the membranes incubated in secondary antibodies coupled to peroxidase (Cwbiotech, China). Finally, the signals were detected by an enhanced chemiluminescence kit (7seapharmtech, China).

### Immunofluorescent staining

For immunofluorescent staining, single mandibular hemi-sections were fixed in 4% paraformaldehyde overnight at 4 °C, decalcified with 10% EDTA (pH 7.0) in shaking incubators. The decalcified solution was refreshed every day for 2 weeks. The samples were embedded in Optimal Cutting Temperature (OCT, Leica) and sectioned into 10-μm sections. Then, the sections were washed with PBS, incubated with 0.03% triton-100 for 10 min at room temperature, and blocked with PBS containing 1% BSA for 30 min at room temperature. Then, the sections were incubated overnight with primary antibodies to p-GSK3β (1:100, Cell signaling technology, 9323), and active β-catenin (1:100, Millipore, 05-665) at 4 °C respectively, and subsequently incubated with fluorescent secondary antibodies (1:200) for 2 h at room temperature. The positive cells were examined under a laser scanning confocal microscope (Olympus FluoViem FV 1000, Tokyo, Japan).

### Micro-computed tomography analysis

The micro-CT scans of single mandibular hemi-sections were performed at the molar level using a GE micro-CT system (GE, USA). X-ray source was set at 80 kV, and 80-μA microfocus. Three-dimensional images were reconstructed, and data analysis was performed by GEHC MicroView analysis software. The single mandibular regions and sagittal planes were chosen for comparison.

### Lentiviral vector construction and transduction

To upregulate ALPL, ALPL was amplified from cDNA of human SEHD by PCR. Then, the PCR product was digested with the restriction enzymes BamH I and Xho I and then inserted into the pLenti 6.3 vector (Invitrogen), named pLenti-ALPL. For downregulation ALPL, the shRNA sequences for targeting human ALPL were inserted in the pLko.1 vector (Invitrogen). The lentivirus contained with pLenti-ALPL or ALPL shRNA were produced by co-transfecting 293T cells with the transfer lentiviral vector and two packaging vectors (psPAX2 and pMD2.G). The primers used to construct the lentiviral vectors of ALPL are listed in Supplementary Table 2.

### β-catenin siRNA transduction

To silence β-catenin expression in HPP DSPCs, β-catenin siRNA (Ribobio, China) were transfected into HPP DSPCs at a final concentration of 50 nM using riboFECT™ CP (Ribobio, China). NC siRNA was transfected as negative control. The silence efficiency on β-catenin was examined after transduction 48 h with β-catenin siRNA by RT-PCR and Western blotting.

### LiCl treatments in vitro

A total of 1 × 10E5 or 2 × 10E5 DPSCs were plated in each well of 12-well or 6-well plates and cultured. After DPSCs reached above 90% confluence, the cells were cultured in odontoblastic differentiation medium containing 10 mM LiCl (Sigma, 213233) or 10 mM NaCl, and the medium was changed three times per week. On days 7 and 21, the cells were harvested and performed with RC-PCR, Western blotting, and Alizarin Red staining as described above.

### LiCl intraperitoneal injection in vivo

A total of 16 4-month-old ALPL^+/−^ mice were randomly divided into NaCl group and LiCl group (*n* = 8 per group). The LiCl group was injected 100 mg/kg LiCl with body weight by intraperitoneal injection every other day for 4 weeks. The control mice received a comparable volume of NaCl. All mice were sacrificed after injection 30 days for teeth analysis by micro-CT, immunofluorescent staining, H&E, and SEM.

### Statistics

The data were presented as the mean ± s.d. All of the experiments were repeated more than three times. Statistical analysis was made using Student’s *t* test or one-way ANOVA, and the *p* < 0.05 was considered significant.

## Results

### Characteristics of the teeth from HPP patients and ALPL^+/−^ mice

**Fig. 1:**
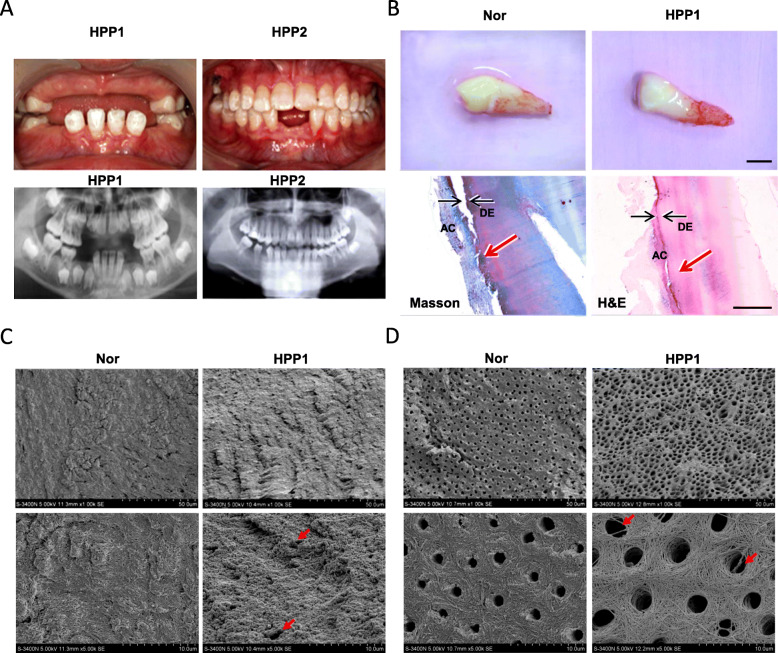
The manifestations of the teeth from patients with HPP. **a** The panoramic radiograph analysis of full dentition of two patients with HPP. **b.** The morphology features of pediatric deciduous cusps from health and HPP1 patient, and histology of tooth structures in HPP1 patient including acellular cementum (AC) and dentin (DE). Upper scale bar = 5 mm, bottom scale bar = 500 mm. **c, d** The surface structures of the cementum (**c**) and ultra-structures of dentinal tubules (**d**) in the tooth from the healthy or HPP patient were detected by the SEM

HPP patients commonly face premature exfoliation teeth caused by dysplasia or aplasia of root or cementum [2, 23, 24]. We have collected two childhood HPP patients reported in our previous study [22]. Two patients displayed premature exfoliation of primary or permanent teeth. According to corresponding panoramic radiograph results, the height of the upper and lower jaws was insufficient, and the permanent dentition featured short roots and delayed eruption in HPP1(Fig. 1a). The alveolar bone height insufficient and short roots were observed in HPP2 (Fig. 1a). Fortunately, one lower cuspid from HPP1 was collected due to severe odontoseisis. The shape of pediatric deciduous cuspid is normal, with no root resorption, and rough root surface (Fig. 1b). Masson staining and H&E staining showed that the cementum layer was apparent thin and uneven surface. In addition, minority root was not covered with cementum, and cementum separated with dentin was observed (Fig. 1b). SEM results further showed that the surface of cementum in HPP1 patient were uneven and rough, and visible dentinal tubules open at root surface, while the surface of the cementum is smooth and homogenous in healthy tooth (Fig. 1c). The dentinal tubules in the HPP1 patient were irregular and low mineralized dentin and were larger lumen with obvious exposure of collagen fiber network than that in the normal deciduous tooth (Fig. 1d).

**Fig. 2:**
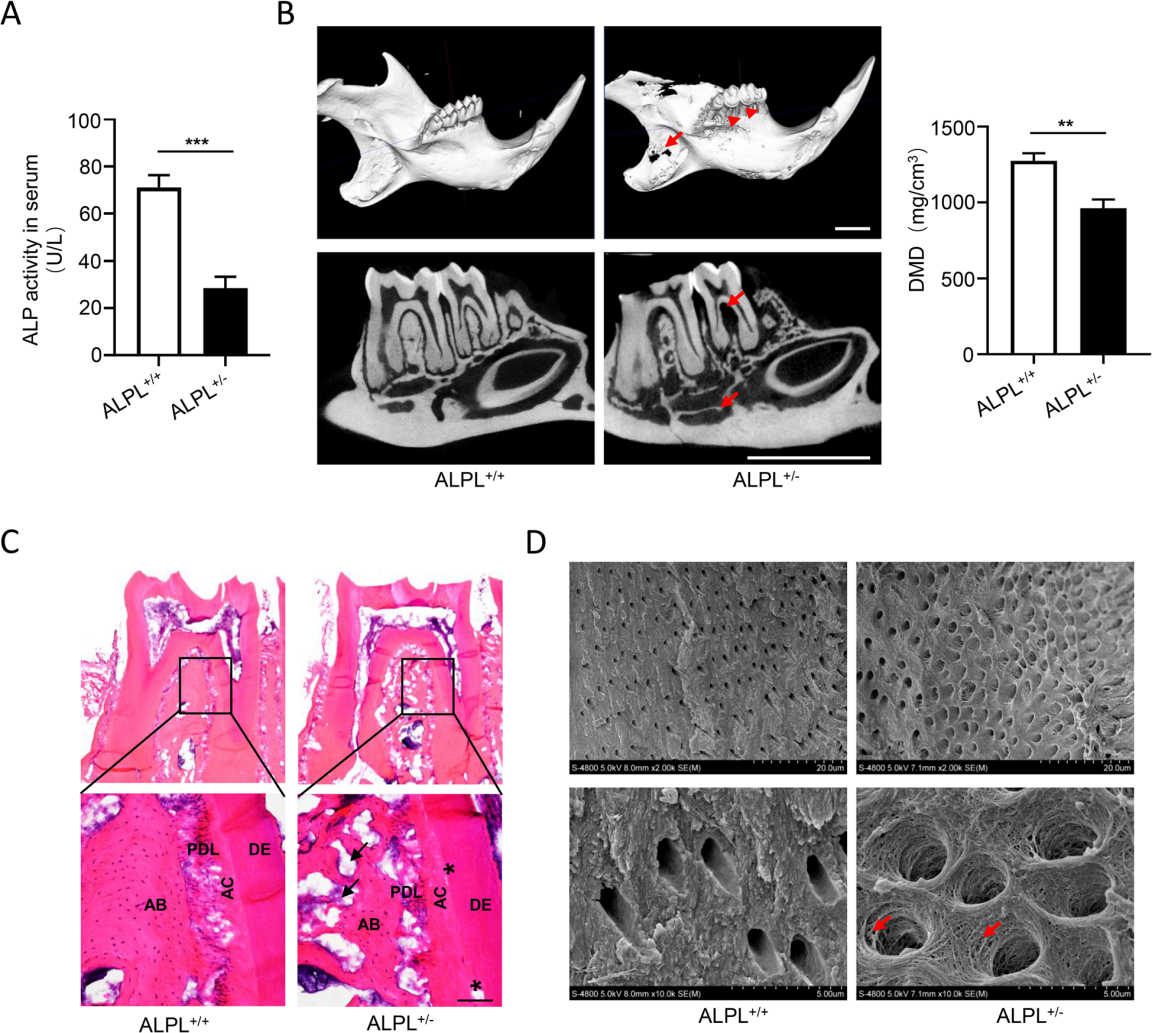
The morphological and histological characters of teeth from ALPL^+/−^ mice. **a** The activity of alkaline phosphatase in serum were examined in WT and ALPL^+/−^ mice. **b** The low jaws including molars, alveolar bone height (arrowheads), bone mass of alveolar bone and mandible bone (arrows), and dentin mineral density (DMD) were analyzed by micro-CT in WT and ALPL^+/−^ mice. Scale bar = 2 mm. **c** The histology of dentoalveolar structures were analyzed including acellular cementum (AC), periodontal ligament (PDL), dentin (DE), and alveolar bone (AB). Scale bar = 100 μm. **d** The ultra-structures of dentinal tubules in the first molars from WT and ALPL^+/−^ mice were analyzed by SEM, the exposure of collagen network as show by the arrows. The data were presented as the mean ± s.d. of triplicate samples and analyzed by Student’s *t* test. **p < 0.01, ***p < 0.001

Previous studies have revealed that ALPL^−/−^ mice suffered severe insufficient mineralization of teeth and skeletal systems [25–27], and skeletal mineralization was significantly impaired in ALPL^+/−^ mice [22, 28]. To investigate tooth defects in HPP, ALPL^+/−^ mice were chosen as animal model, because of ALPL^−/−^ mice die from 14 to 21 days after birth [29]. First, we found the ALP activity in serum of ALPL knockdown mice reduced approximately 60% as compared with that of wild type mice (Fig. 2a). Next, to evaluate the effects of ALPL deficiency on the tooth, micro-CT analysis was performed. The results showed that the alveolar bone height, mandibular bone mass, and dentin mineral density (DMD) dramatically reduced in ALPL^+/−^ mice compared with that in the control group (Fig. 2b). Moreover, ALPL knockdown significantly decreased the alveolar bone mass and led to cementum partially segregated from dentin (Fig. 2c, d). In addition, the dentinal tubules of the molars in ALPL^+/−^ mice exhibited larger lumen with collagen fiber network exposure in contrast to the control group (Fig. 2d), which was observed in the HPP1 patient. Collectively, these results suggested ALPL^+/−^ mice exhibited tooth defects similar to HPP-associated periodontal disorder.

### ALPL mutation impaired cell self-renewal and proliferation rather than cell apoptosis of DPSCs

**Fig. 3:**
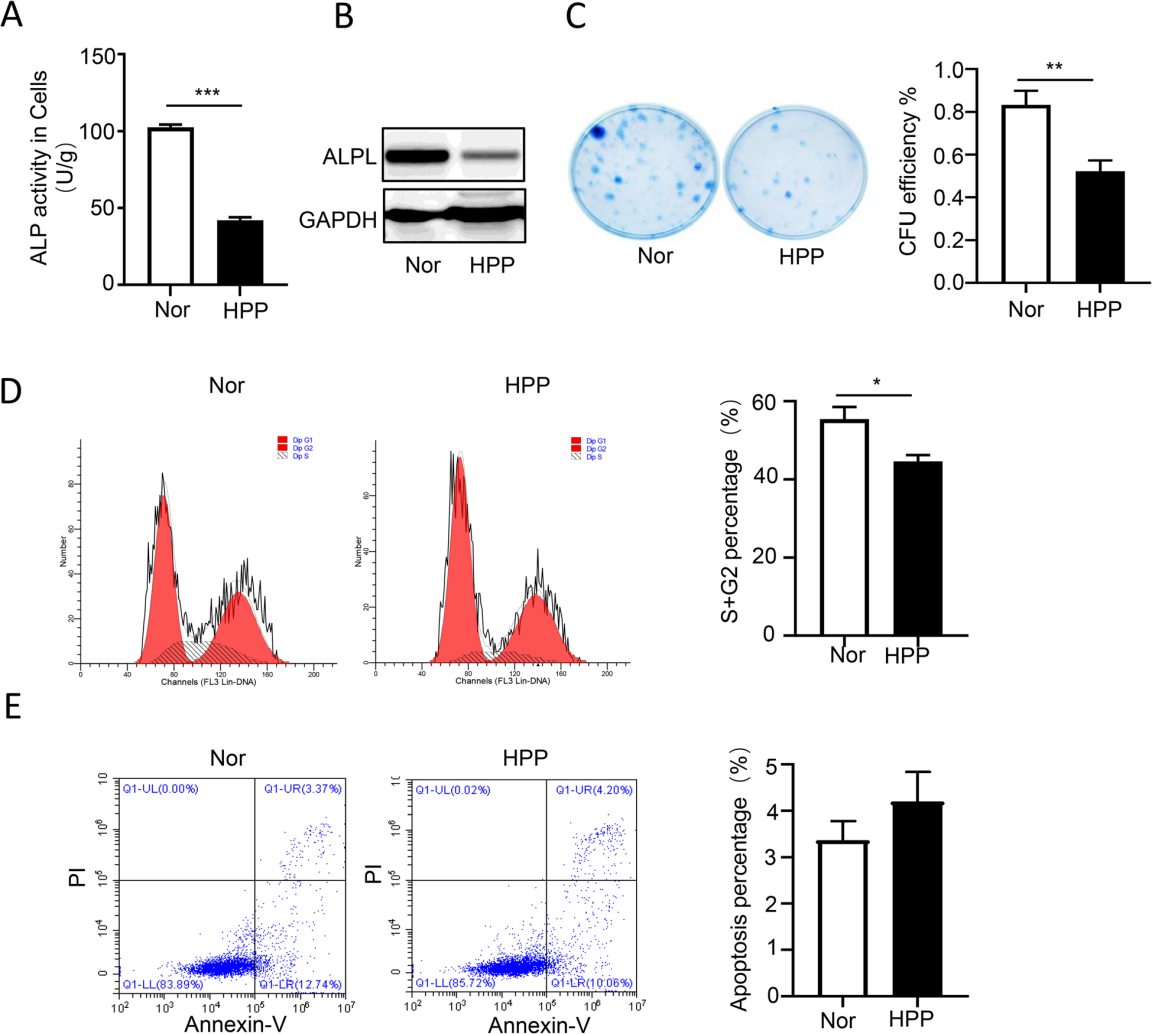
The influence of ALPL mutation on cell proliferation and apoptosis of DPSCs. **a, b** The ALP activities (**a**) and the ALPL expression (**b**) on protein were examined in DPSCs from normal and patient with HPP. **c** The effective CFU of DPSCs in culture medium was compared between control and HPP groups in vitro. **d, e** The cell cycle (**d**) and cell apoptosis (**e**) of DPSCs from normal and HPP patient were performed by flow cytometry analysis. The data were analyzed by Student’s *t* test and presented as the mean ± s.d. of triplicate samples from a representative experiment. *p < 0.05, **p < 0.01, ***p < 0.001

Increasing data provided the evidences that DPSCs cloud be induced to differentiate into odontoblasts and regenerate a dental pulp-dentin like tissue in vivo [13, 14]. To further assess the role of ALPL in teeth development, DPSCs derived from HPP1 patient were isolated and cultured. First, we found that alkaline phosphatase activity and the protein expression of ALPL in HPP group decreased significantly compared with that of the normal group (Fig. 3a). Then, to evaluate the roles of ALPL on cell self-renewal, cell proliferation, and apoptosis, the tests of colony formation, cell cycle, and cell apoptosis were performed. In contrast to normal DPSCs, the colony formation unit (CFU) efficiency significantly impaired (Fig. 3b) and the phage of S + G2 in cell cycle (Fig. 3c) obviously reduced in HPP group, but the difference in cell apoptosis was not observed (Fig. 3d). Our results suggested that ALPL deficiency impaired cell self-renewal and proliferation of DPSCs.

### ALPL regulates the odontoblastic differentiation of DPSCs

**Fig. 4:**
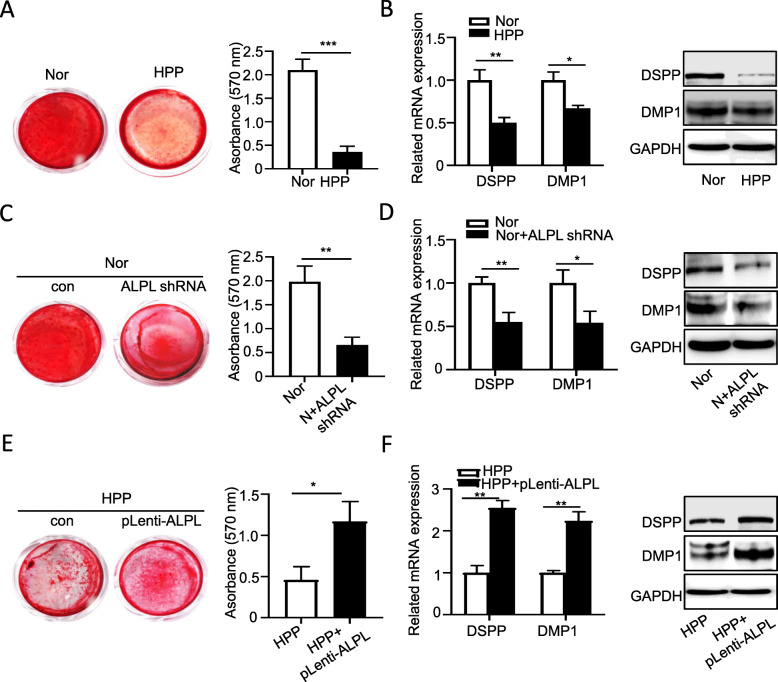
ALPL regulates the odontoblastic differentiation of DPSCs. **a** Alizarin Red staining was performed in DPSCs from the health and HPP groups after cultured in odontoblastic medium for 3 weeks. **b** The expression of DMP1 and DSPP was examined on gene and protein level in DPSCs from the health and HPP groups after cultured in odontoblastic medium for 7 days (*n* = 3). To evaluate whether ALPL could regulate DPSC differentiation into odontoblasts, we upregulated or downregulated the expression of ALPL in HPP DPSCs with pLenti-ALPL or normal DPSCs with pLko.1-ALPL shRNA lentiviral transduction. **c**, **e** Alizarin Red staining was performed in DPSCs from health and health with pLko.1-ALPL shRNA (**c**), or DPSCs from HPP and HPP with pLenti-ALPL (**e**) after cultured in odontoblastic medium for 3 weeks. **d**, **f** The expression of DMP1 and DSPP was examined on gene and protein level in DPSCs from health and health with pLko.1-ALPL shRNA (**d**), or DPSCs from HPP and HPP with pLenti-ALPL (**f**) after cultured in odontoblastic medium for 7 days (*n* = 3). The data were presented as the mean ± s.d. of triplicate samples and analyzed by Student’s *t* test. *p < 0.05, **p < 0.01, ***p < 0.001

Given that impaired mineralization and exposure of collagen fiber network on dentinal tubules were observed both in HPP patient and ALPL^+/−^ mice, we next evaluated the capacity of DPSCs from HPP differentiation into odontoblasts in vitro. The results of Alizarin Red staining showed that the potential odontoblastic differentiation capacity of HPP DPSCs significantly impaired (Fig. 4a), along with decreased expression of odontoblast-associated genes and proteins, DSPP and DMP1 (Fig. 4b). To further validate the role of ALPL in regulatory odontoblastic differentiation, we upregulated or downregulated the expression of ALPL in HPP DPSCs with plenti-ALPL lentivirus or normal DPSCs with plko.1-ALPL shRNA lentivirus (sFig. 2). The odontoblastic differentiation of normal DPSCs was inhibited by the downregulation of ALPL (Fig. 4c, d), mimicking the characteristic of HPP DPSCs, whereas recovery ALPL expression in HPP DPSCs remarkably promoted odontoblastic differentiation (Fig. 4e, f) as evidenced by Alizarin Red staining, and expressions of odontoblast associated genes and proteins. Therefore, our results suggest that ALPL could be essential for DPSCs maintaining odontoblastic differentiation property.

### ALPL regulates DPSC differentiation into odontoblasts by controlling canonical Wnt signaling pathway

**Fig. 5:**
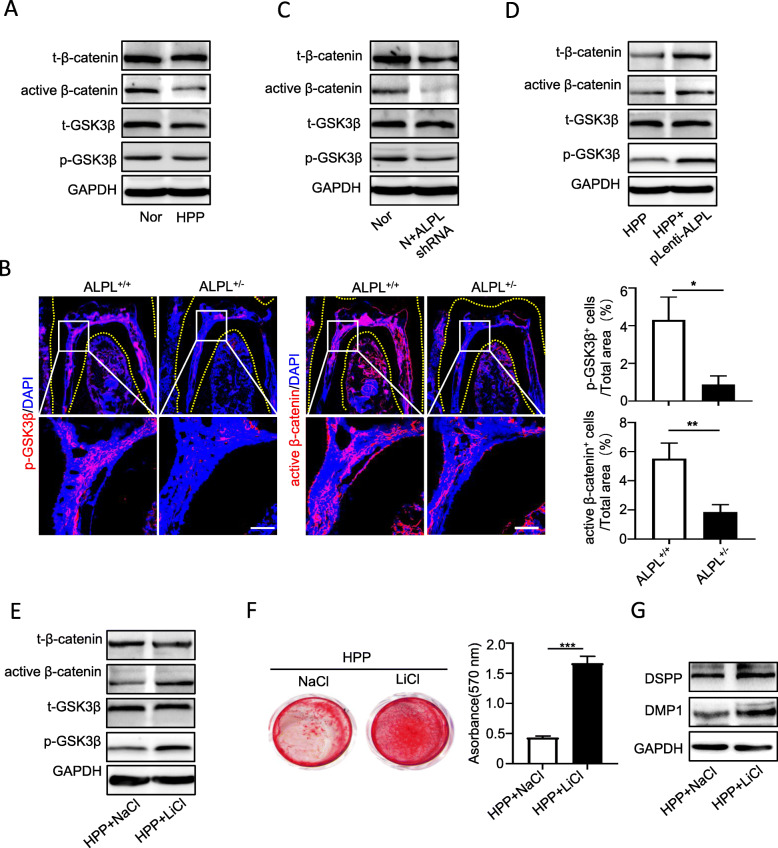
LiCl restored odontoblastic differentiation of HPP DPSCs by promoting canonical Wnt pathway activation. **a** Canonical Wnt pathway-related key proteins, including total and phosphorylation of GSK3β, and total and active β-catenin in DPSCs from health and HPP groups were examined by Western blotting. **b** Immunofluorescence staining of p-GSK3β and active β-catenin were performed in the first molars of ALPL^+/+^ and ALPL^+/−^ mice. Scale bar = 100 μm. **c–e**. The expression of total and phosphorylation of GSK3β, and total and active β-catenin in DPSCs from health and health with pLko.1-ALPL shRNA (**c**), DPSCs from HPP and HPP with pLenti-ALPL (**d**), or DPSCs from HPP and HPP treated with LiCl (**e**). **f** Alizarin Red staining was performed in DPSCs from HPP and HPP treated with LiCl after cultured in odontoblastic medium for 3 weeks. **g** The protein expression of DMP1 and DSPP were examined in DPSCs from HPP and HPP treated with LiCl after cultured in odontoblastic medium for 7 days. The data were presented as the mean ± s.d. of triplicate samples and analyzed by Student’s *t* test. *p < 0.05, **p < 0.01, ***p < 0.001

Our previous study has demonstrated that ALPL deficiency-induced dysregulation of the phosphorylation of intracellular signaling proteins in MSCs [22]. We found that the protein levels of phosphorylation of glycogen synthase kinase 3β (p-GSK3β), and active β-catenin in HPP DPSCs significantly decreased compared with that in healthy cells (Fig. 5a). In addition, Gli1 and phosphorylation of AMP-activated protein kinase α (AMPKα) also decreased in HPP DPSCs, but not extracellular signal-regulated kinase1/2 (p-ERK1/2) (sFig. 3). Considering that GSK3β-mediated canonical Wnt signaling pathway is a key signaling pathway for regulating tooth development [30, 31], the expression of p-GSK3β and active β-catenin in the first molars were examined by immunofluorescent staining. Notably, in contrast to the wild-type group, the expression of p-GSK3β and active β-catenin significantly decreased especially in the dental pulp and alveolar bone in ALPL^+/−^ mice as showed in immunofluorescence staining (Fig. 5b).

Then, to evaluate whether ALPL could regulate p-GSK3β and active β-catenin expression, ALPL were downregulated in healthy DPSCs or upregulated in HPP DPSCs, respectively. The Western blotting results showed that downregulation of ALPL in normal cells significantly inhibited p-GSK3β and active β-catenin expression, while ALPL overexpression effectively promoted the expression of p-GSK3β and active β-catenin in HPP DPSCs (Fig. 5c, d). These results suggested that ALPL deficiency could disrupt canonical Wnt pathway activation and then impair DPSC differentiation into odontoblasts.

To resolve above question, the small molecule LiCl, which is an agonist for high effective activating canonical Wnt signaling pathway by inhibited GSK3β activity [32, 33], were used to treat HPP DPSCs. As we expected, the expression of phosphorylation of GSK3β and active β-catenin increased dramatically in HPP DPSCs with LiCl treatment (Fig. 5e). Importantly, in contrast to the control group, the odontoblastic differentiation was prominently improved in HPP DPSCs after treated with LiCl by enhanced Alizarin Red staining and the protein expression of DSPP and DMP1 (Fig. 5f, g).

**Fig. 6:**
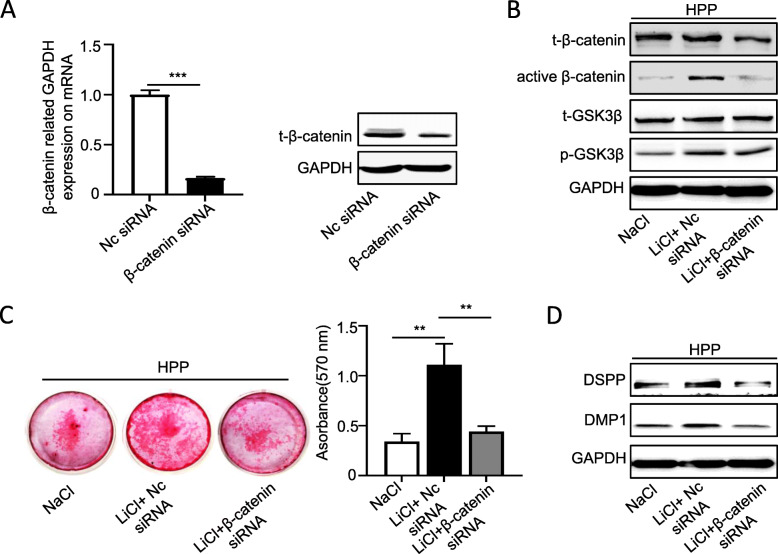
LiCl rescued odontoblastic differentiation of HPP DPSCs were blocked by β-catenin siRNA. **a**, **b** The β-catenin silence efficiency was examined by RT-PCR (**a**) and Western blotting (**b**) in HPP DPSCs with β-catenin siRNA transfection for 48 h (*n* = 3). **c** The expression of t-GSK3β and p-GSK3β, and the total and active β-catenin were examined by Western blotting in HPP DPSCs from the NaCl control, LiCl+Nc siRNA, and LiCl+β-catenin siRNA groups, which were cultured in odontoblastic medium for 7 days. **d**, **e** To evaluate LiCl rescue odontoblastic differentiation of HPP DPSCs by canonical Wnt pathway, the cells were treated with LiCl meanwhile transected with β-catenin siRNA. The odontoblastic differentiation was examined by Alizarin Red staining (**d**) after in odontoblastic medium for 3 weeks, and Western blotting (**e**) after in odontoblastic medium for 7 days. The data were presented as the mean ± s.d. of triplicate samples and analyzed by Student’s *t* test or one-way ANOVA. **p < 0.01, ***p < 0.001

To further demonstrate that LiCl rescued the odontoblastic differentiation of HPP DPSCs by activating canonical Wnt pathway, these cells were treated with LiCl meanwhile interfered with β-catenin siRNA to block canonical Wnt pathway activation. The RT-PCR and Western blotting results showed that β-catenin siRNA strong silenced the expression of β-catenin in HPP DPSCs (Fig. 6a). Then, we found that the increased expression of active β-catenin, but not p-GSK3β, in HPP DPSCs with LiCl treatment was inhibited by β-catenin siRNA (Fig. 6b). In addition, LiCl-improved odontoblastic differentiation of HPP DPSCs was almost blocked by silence β-catenin expression according to the results of Alizarin Red staining and Western blotting (Fig. 6c, d). Collectively, these results indicated that reactivated GSK3β to β-catenin signaling cascade cloud improve odontoblastic differentiation of HPP DPSCs.

### LiCl treatment ameliorates insufficient mineralization of dentin and mandible in ALPL^+/−^ mice

**Fig. 7:**
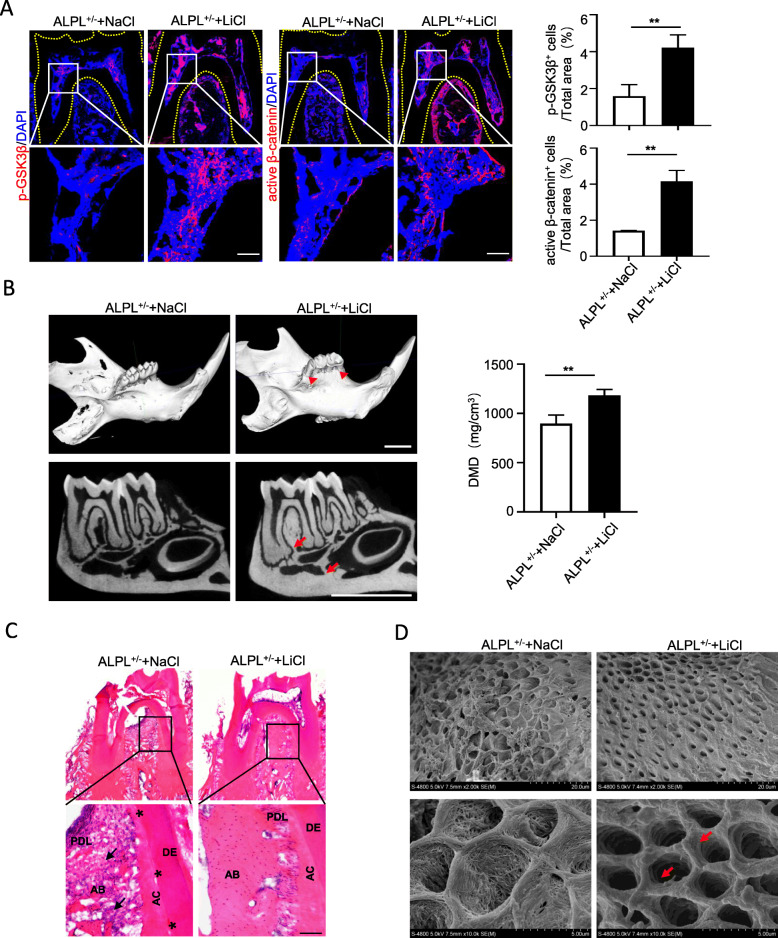
GSK3β rephosphorylation ameliorated tooth associated defects in ALPL^+/−^ mice with LiCl treatment. **a** Immunofluorescence staining of p-GSK3β and active β-catenin was performed in the first molars of ALPL^+/−^ mice and ALPL^+/−^ mice with LiCl treatment. Scale bar = 100 μm. **b** The micro-CT analysis of the low jaws including the molars, alveolar bone height (arrowheads), bone mass of alveolar bone and mandible bone (arrows), and DMD was performed in ALPL^+/−^ mice and ALPL^+/−^ mice treated with LiCl. Scale bar = 2 mm. **c** The histology of dentoalveolar structures including acellular cementum (AC), periodontal ligament (PDL), dentin (DE), and alveolar bone (AB) was analyzed in the first molars of ALPL^+/−^ mice and ALPL^+/−^ mice treated with LiCl. Scale bar = 100 μm. **d** SEM analysis the ultra-structures of dentinal tubules in the first molars from ALPL^+/−^ mice and ALPL^+/−^ mice treated with LiCl, and the dentinal tubules with exposure of collagen network of ALPL^+/−^ mice were significantly improved after LiCl therapy as show by the arrows. *n* = 8 mice per group. The data were presented as the mean ± s.d. of triplicate samples and analyzed by Student’s *t* test. **p < 0.01

**Fig. 8:**
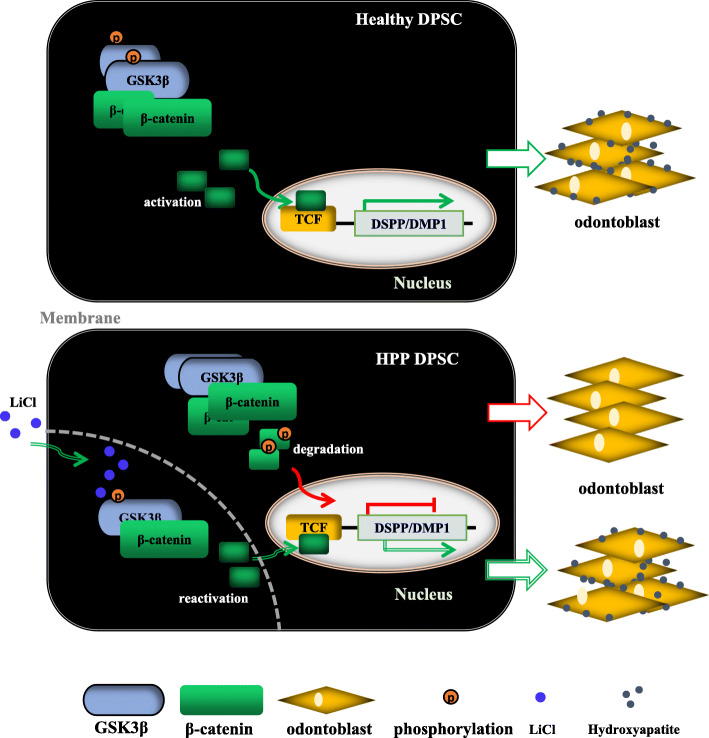
Schematic diagram depicts how ALPL deficiency impairs DPSC differentiation into odontoblasts and a therapeutic method. In Healthy DPSC, ALPL is essential for regulating canonical Wnt signal pathway activation, which increases the expression of odontoblast-associated gene, DSPP and DMP1, and subsequently promotes odontoblastic differentiation of DPSC. In HPP DPSC, ALPL deficiency caused by ALPL mutation, disrupted intracellular GSK3β to β-catenin signaling cascade, then impaired odontoblastic differentiation of DPSC. While LiCl treatment can rescue the impairment of odontoblastic differentiation of HPP DPSC through reactivating intracellular GSK3β to β-catenin signaling transduction

Next, to evaluate whether LiCl plays a therapeutic benefit on improving insufficient mineralization of the molars and mandible in ALPL knockout mice, the mice were administered LiCl every other day for 1 month. First, we observed the protein levels of p-GSK3β and active β-catenin increased significantly in the dental pulp and alveolar bone of ALPL^+/−^ mice after LiCl treatment 1 month, compared with those in the NaCl group (Fig. 7a). Notably, micro-CT analysis confirmed that ALPL^+/−^ mice treated with LiCl displayed high alveolar bone height, increased bone mass of mandibular bone and alveolar bone, and DMD compared with those of the NaCl group (Fig. 7b). Further, the H&E staining showed that LiCl injection increased the alveolar bone mass and improved segregation between dentin and cementum in ALPL knockdown mice (Fig. 7c). Meanwhile, the dentinal tubules with the larger lumen and collagen fiber network exposure in the molars of ALPL^+/−^ mice were also ameliorated after LiCl treatment (Fig. 7d). In summary, our results provided the convincing proof for the mechanism that ALPL deficiency inhibited intracellular GSK3β to β-catenin signaling cascade leading to impairment of odontoblastic differentiation in DPSCs. While LiCl treatment could rescue such impairment through reactivating canonical Wnt pathway (Fig. 8).

## Discussion

Tooth-associated defects, such as short roots with thin dentin, lack of acellular cementum, and alveolar bone loss, are the most common clinical manifestations of patients with HPP [1, 23, 24, 34, 35], and these phenotypes were also found in ALPL knockout mice or condition ALPL knockout mice [11, 27, 36]. However, the mechanism of dysplasia or aplasia of the teeth caused by loss of function of ALPL remains elusive. In this study, our goals were to evaluate the influence of ALPL mutation on bio-behaviors of DPSCs and reveal the potential mechanism on cellular signal transduction. Here, we provided the evidence that ALPL deficiency inhibited cell proliferation and odontoblastic differentiation of DPSCs from HPP patient. Furthermore, ALPL gene mutation caused GSK3β maintaining low phosphorylation status, and prevented β-catenin translocation into nucleus and inhibited activation of canonical Wnt signaling pathway, which is one of main signaling pathways for controlling lineage differentiation of MSCs [19, 37]. Notably, LiCl treatment reactivated intracellular GSK3β to β-catenin signaling cascade in DPSCs or dental pulp and restored impairment of odontoblastic differentiation, then ameliorated teeth and periodontal-related disorders in ALPL^+/−^ mice.

Previous studies showed the accumulation of pyrophosphate (PPi), one of substrates of ALPL, in culture medium-impaired mineralization of dental pulp cells from HPP patients [17, 38]. However, researchers have not elucidated whether ALPL induces a series of intracellular changes. Here, we provided evidences that ALPL deficiency disrupted GSK3β to β-catenin signaling cascade and then inhibited expression of odontoblastic-associated genes, DMPP and DMP1, in DPSCs from HPP patient. Traditional knowledge indicates that ALPL is a downstream gene of canonical Wnt pathway [20, 39, 40] and plays main function on extracellular matrix mineralization of osteoblast, odontoblast, and ameloblast. Nevertheless, our results indicated that ALPL might be as a distinct regulator to control cellular signals cascade in MSCs. It may provide useful information for understanding ALPL mutations leading to tooth dysplasia.

Currently, enzyme replacement therapy with asfotase alfa was recognized as the only effective clinical treatment option for HPP. Increasing clinical data demonstrated that the most severe forms of HPP (prenatal and infantile) with asfotase alfa treatment significantly increased in bone mineral density and improved in respiratory and motor function [9, 41, 42]. One study recently reported about the dental status of HPP patients with asfotase alfa treatment. In their study, the author showed that enzyme replacement therapy significant decreased the mobility of the primary tooth in some cases [12], but a clinically defined improvement was also observed in some patients despite with long-term asfotase alfa treatment. The efficacy evaluation of the asfotase alfa on dental defects in HPP patients is very limited based on the current clinical data. Thus, prior to asfotase alfa therapy for dentist, the potential risks, effective treatment, and also expensive cost should be considered.

In the present study, we observed systemic LiCl injections effectively improved mineralization of dentinal tubules, but also increased height and bone mass of alveolar bone, and DMD in ALPL knockdown mice. We could not exclude whether other types of cells in periodontal tissues were also affected by systemic LiCl injections. Indeed, periodontal ligament stem cells derived from tooth and BMSCs from the jaw are also important for the tooth development and functional maintenance [43]. Despite that our previous works demonstrated LiCl treatment improved bone mass loss of femora, it might explain why systemic LiCl injections also increased bone mass of the alveolar bone in ALPL^+/−^ mice. It still needs to be evaluated whether ALPL deficiency disrupts GSK3β/β-catenin cascade, and LiCl exerts therapeutic benefits in these cells. Therefore, it urgently needs to evaluate LiCl therapeutic benefits in dental stem cells and tooth defects in ALPL knockdown mice and the regulatory relationship between ALPL and GSK3β in the following works, and ultimately, these works may inform efforts at improvement tooth defects in HPP patients.

## Conclusions

Here, our study indicated that ALPL might be as a distinct regulator to control intracellular GSK3β to β-catenin signals cascade and subsequently regulate DPSC differentiation into odontoblast. ALPL deficiency impaired odontoblastic differentiation of DPSCs, while LiCl treatment could rescue such impairment through rephosphorylation of GSK3β. This work may provide useful information for understanding how ALPL mutations leading to tooth dysplasia and, ultimately, may inform efforts at improvement tooth defects in patients with HPP.

## Supplementary Information

**Additional file 1: sFig. 1** identification of DPSCs phenotype by flow cytometer. The makers of DPSCs, including CD29, CD44, CD105, CD34, CD45, and Stro-1, were examined by flow cytometric cytometer in health and patient with HPP.

**Additional file 2: sFig. 2** The silence and overexpression efficiency of ALPL. **a** The expression of ALPL protein were examined by western blotting in health DPSCs and health DPSCs with pLko.1-ALPL shRNA lentiviral transduction for 48 h. **b** The expression of ALPL protein were examined by western blotting in HPP DPSCs and HPP DPSCs with pLenti-ALPL lentiviral transduction for 48 h.

**Additional file 3: sFig. 3** The expression of signaling proteins in DPSCs. The expression of signaling proteins, including p-ERK, p-AMPK, Gli1, and TGFβ, were examined by western blotting in DPSCs from health and HPP groups.

**Additional file 4: Supplementary Table 2**. Primers sequences used to construct the ALPL lentiviral vectors. **Supplementary Table 1**. Primers sequences for Real-Time PCR assay.

## Data Availability

All datasets used and/or analyzed during the current study are available from the corresponding author on reasonable request.

## Abbreviation

ALPL: Liver/bone/kidney-type alkaline phosphatase
HPP: Hypophosphatasia
DPSC: Dental pulp stem cells
t-GSK3β: Total glycogen synthase kinase 3
DSPP: Dentin sialophosphoprotein
DMP1: Dentin matrix acidic phosphoprotein 1
p-GSK3β: Phosphorylation of glycogen synthase kinase 3 beta
micro-CT: Micro-computed tomography
RT-PCR: Real-time polymerase chain reaction
