# Supplementary figures and images for: GSK3β rephosphorylation rescues ALPL deficiency-induced impairment of odontoblastic differentiation of DPSCs
Source: Stem Cell Res Ther. 2021 Apr 6;12:225. doi: 10.1186/s13287-021-02235-7 (PMC8022410; doi:10.1186/s13287-021-02235-7)

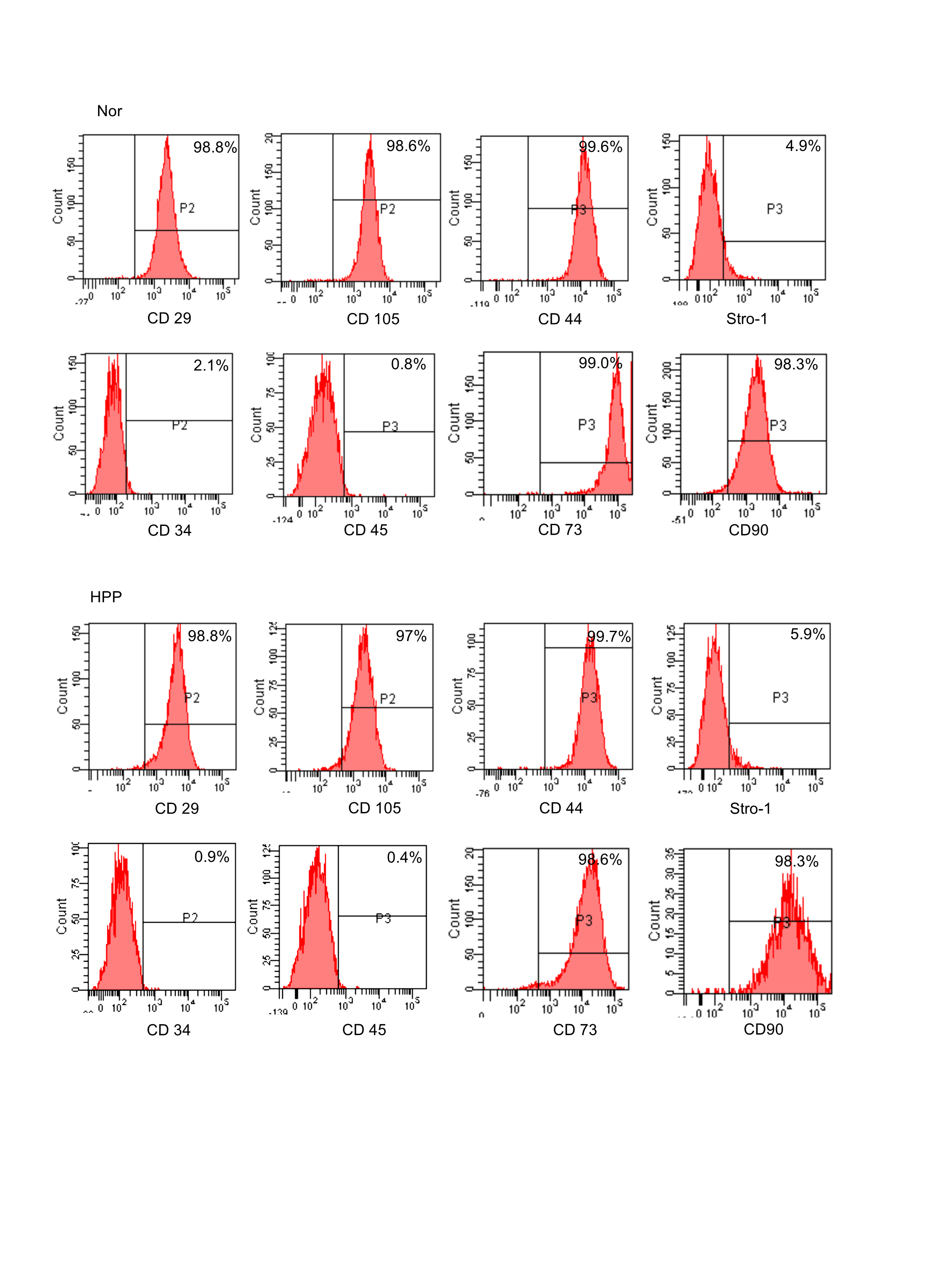

Supplement: Supplementary file 1 — Additional file 1: sFig. 1 identification of DPSCs phenotype by flow cytometer. The makers of DPSCs, including CD29, CD44, CD105, CD34, CD45, and Stro-1, were examined by flow cytometric cytometer in health and patient with HPP. [file 13287_2021_2235_MOESM1_ESM.tif]

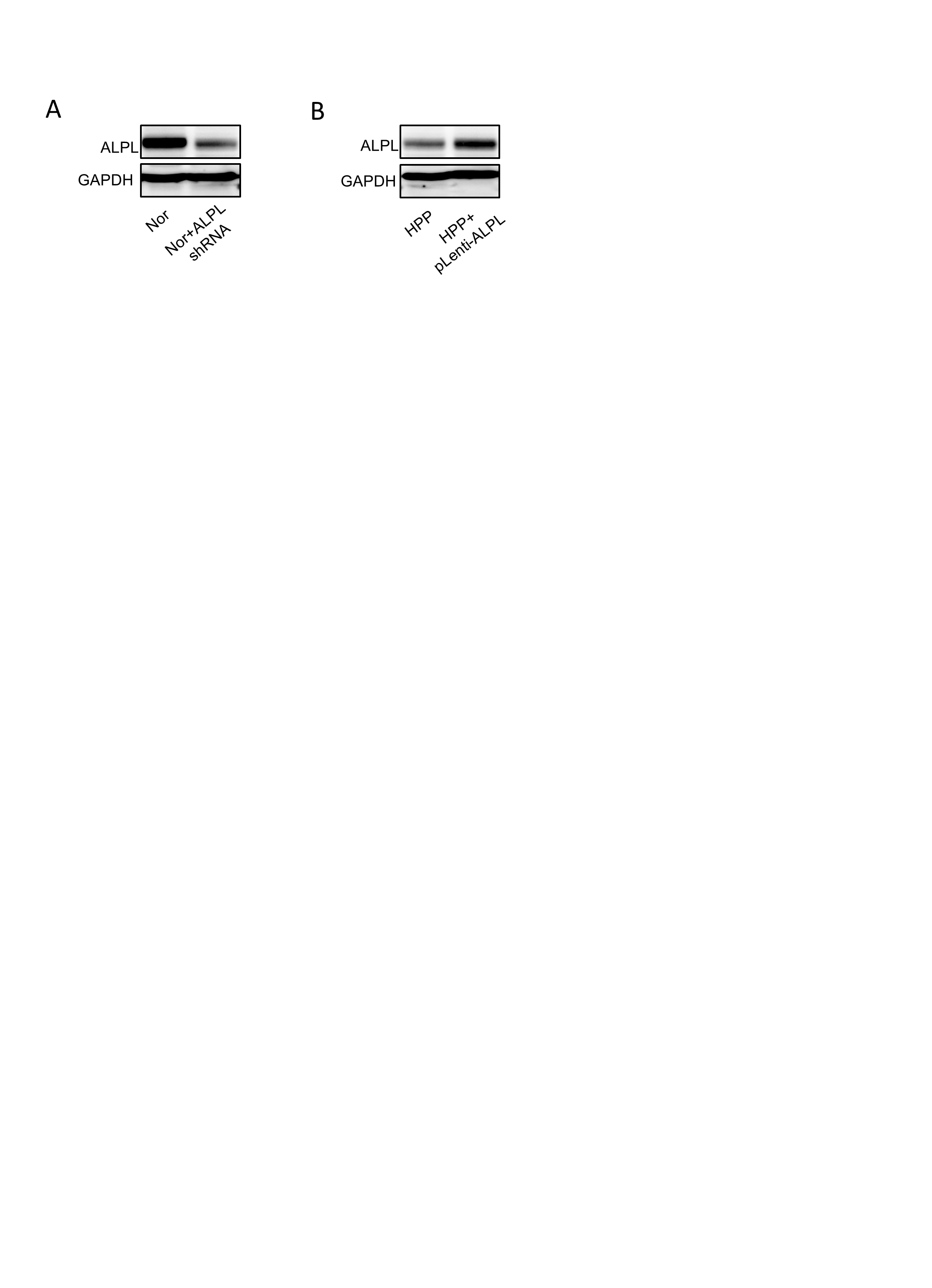

Supplement: Supplementary file 2 — Additional file 2: sFig. 2 The silence and overexpression efficiency of ALPL. a The expression of ALPL protein were examined by western blotting in health DPSCs and health DPSCs with pLko.1-ALPL shRNA lentiviral transduction for 48 h. b The expression of ALPL protein were examined by western blotting in HPP DPSCs and HPP DPSCs with pLenti-ALPL lentiviral transduction for 48 h. [file 13287_2021_2235_MOESM2_ESM.tif]

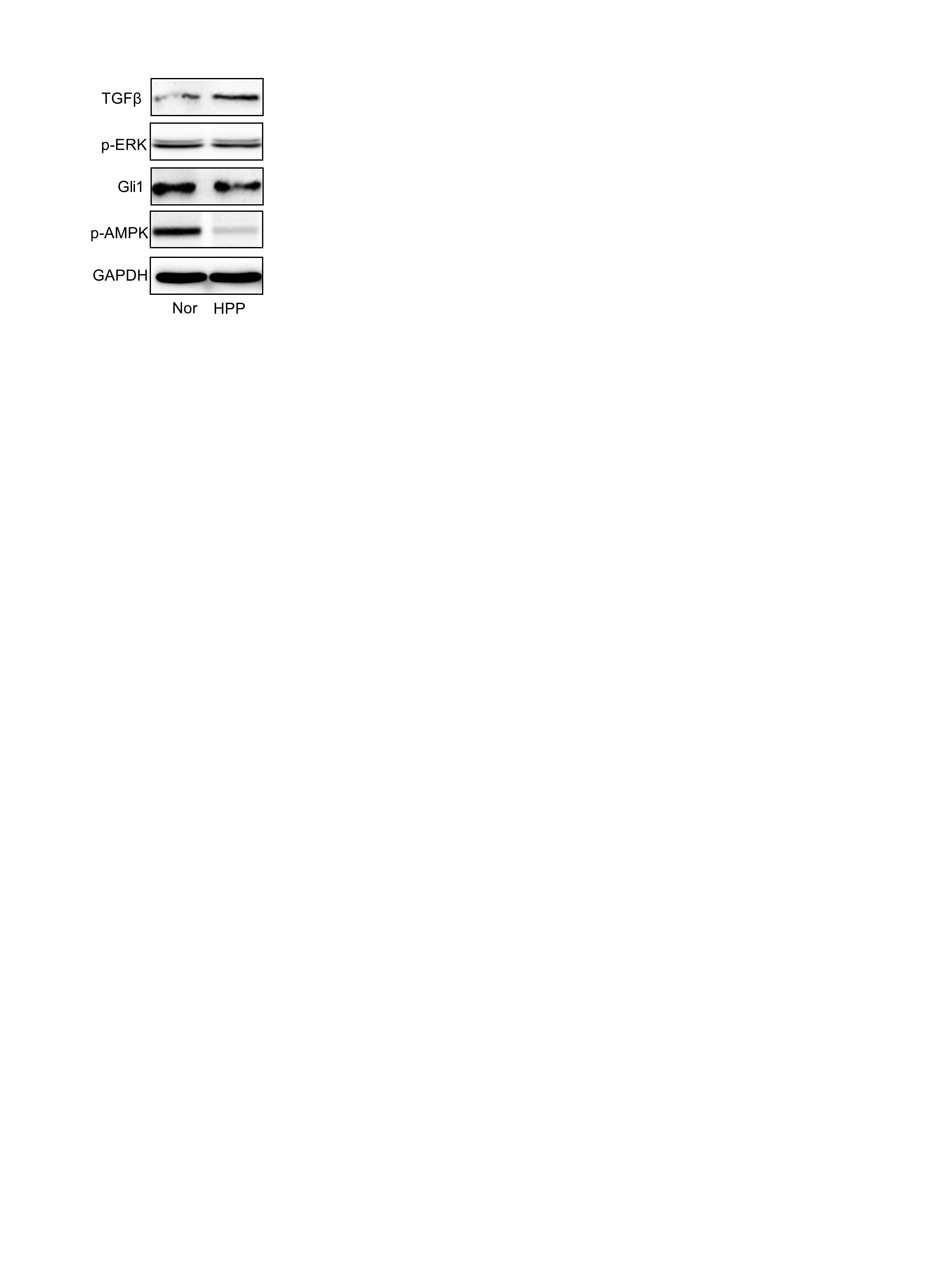

Supplement: Supplementary file 3 — Additional file 3: sFig. 3 The expression of signaling proteins in DPSCs. The expression of signaling proteins, including p-ERK, p-AMPK, Gli1, and TGFβ, were examined by western blotting in DPSCs from health and HPP groups. [file 13287_2021_2235_MOESM3_ESM.tif]
