# Supplementary material for: GSK3β rephosphorylation rescues ALPL deficiency-induced impairment of odontoblastic differentiation of DPSCs
Source: Stem Cell Res Ther. 2021 Apr 6;12:225. doi: 10.1186/s13287-021-02235-7 (PMC8022410; doi:10.1186/s13287-021-02235-7)
Supplement: Supplementary file 4 — Additional file 4: Supplementary Table 2. Primers sequences used to construct the ALPL lentiviral vectors. Supplementary Table 1. Primers sequences for Real-Time PCR assay. [file 13287_2021_2235_MOESM4_ESM.docx]

Supplementary Table 1.

Primers sequences for Real-Time PCR assay

| Gene name | Forward primer (5’ to 3’) | Reverse primer (5’ to 3’) |
| --- | --- | --- |
| DMP1 | AGCCATTCTGAGGAAGACGA | TGTTGTGATAGGCATCAACTGTTA |
| DSPP | GCATTCAGGGACAAGTAAGCA | CTTGGACAACAGCGACATCCT |
| β-catenin | TTGAAAATCCAGCGTGGACA | TCGAGTCATTGCATACTGTC |
| GAPDH | GCACCGTCAAGGCTGAGAAC | TGGTGAAGACGCCAGTGGA |

Supplementary Table 2.

Primers sequences used to construct the ALPL lentiviral vectors

| Vector name | Sequences (5’ to 3’) |
| --- | --- |
| pLko.1-ALPL shRNA | F-CCGGGCAGTATGAATTGAATCGGAACTCGAGTTCCGATTCAATTCATACTGCTTTTTG  R-AATTCAAAAAGCAGTATGAATTGAATCGGAACTCGAGTTCCGATTCAATTCATACTGC |
| pLenti-ALPL | F-ACTGGATCCCAGCGAGGGACGAATCTCAGG  R-TATCTCGAGGGGGAGCTGGCTGTCCATTG |
